# Comparative Transcriptomic Analysis Provides Insights into PSK-α-Mediated Regulation of Diosgenin Biosynthesis in *Trigonella foenum-graecum*

**DOI:** 10.3390/molecules31173046

**Published:** 2026-08-30

**Authors:** Chuanjia Xu, Hao Zhan, Malakkhanim Mehraliyeva, Haoran Liu, Mengmeng Zheng, Changfu Li, Yansheng Zhang

**Affiliations:** 1 Shanghai Key Laboratory of Bio-Energy Crops, Synthetic Biology Research Center, School of Life Sciences, Shanghai University, Shanghai 200444, China; xuchuanjia@shu.edu.cn (C.X.); 2951167064@shu.edu.cn (H.Z.); malakmehraliyeva@gmail.com (M.M.); liuhaoran001@shu.edu.cn (H.L.); mmz@shu.edu.cn (M.Z.); changfuli@shu.edu.cn (C.L.); 2 School of Environmental and Chemical Engineering, Shanghai University, Shanghai 200444, China

**Keywords:** *Trigonella foenum-graecum*, PSK-α, diosgenin biosynthesis, PSK signaling pathway, transcription factor

## Abstract

Diosgenin is an important steroidal sapogenin widely used for the industrial production of steroidal drugs. Phytosulfokine-α (PSK-α) is a sulfated pentapeptide hormone known to regulate plant growth, development, and stress responses. However, its role in the regulation of diosgenin biosynthesis remains unclear. Fenugreek seedlings were treated with PSK-α to investigate its effects on diosgenin biosynthesis through metabolite profiling and comparative transcriptomic analyses. PSK-α induced a dynamic response in diosgenin accumulation, characterized by an initial decrease during the early stage of treatment followed by a gradual increase, ultimately resulting in 61.4% and 72.1% increases in diosgenin accumulation after 10 days and 15 days, respectively, compared with the corresponding random pentapeptide control. Comparative transcriptomic analysis between PSK-α-treated seedlings and the corresponding controls at 5 and 15 days identified key genes or transcript isoforms involved in diosgenin biosynthesis, PSK signaling and MAPK signaling pathways. Meanwhile, five candidate genes involved in diosgenin biosynthesis and PSK signaling exhibited expression patterns consistent with the dynamic accumulation pattern of diosgenin. To further dissect the regulatory network, co-expression network analysis between these 28 candidate genes involved in diosgenin biosynthesis and PSK signaling and 1407 transcription factor-encoding genes identified candidate transcription factors potentially involved in the coordinated regulation of diosgenin biosynthesis and PSK signaling. This study suggests that PSK-α may contribute to enhanced diosgenin accumulation through coordinated transcriptional regulation of diosgenin biosynthesis and PSK signaling pathways, providing new insights into the regulation of plant secondary metabolism by peptide hormones.

## 1. Introduction

Diosgenin is a spirostanol steroidal sapogenin widely distributed among plant species of Dioscoreaceae, Fabaceae and Zygophyllaceae, with major natural sources including the monocotyledonous species *Dioscorea zingiberensis*, an important industrial source of diosgenin, and the medicinal dicotyledonous legume *Trigonella foenum-graecum* [1]. In plants, it functions primarily as the aglycone of steroidal saponins and has been implicated in responses to biotic and abiotic stresses as well as defense-related processes [2,3]. Beyond its physiological roles, diosgenin exhibits diverse pharmacological activities, including anti-tumor, hypoglycemic, anti-inflammatory, and cardioprotective effects [4,5,6,7,8], and serves as an important industrial precursor for the synthesis of steroidal drugs such as progesterone, testosterone, and hydrocortisone [9]. Because of its pharmaceutical importance and industrial value, considerable efforts have been devoted to elucidating the biosynthesis pathway and regulatory mechanisms of diosgenin. Among diosgenin-producing species, fenugreek (*T. foenum-graecum*) has emerged as a valuable model system due to its short growth cycle, ease of cultivation, and high accumulation of bioactive metabolites [10,11].

Previous studies have demonstrated that treatment with exogenous elicitors is an effective strategy for elucidating the regulatory mechanisms underlying plant secondary metabolism and identifying key regulatory factors [12,13,14]. For example, methyl jasmonate (MeJA) treatment significantly promotes diosgenin accumulation in fenugreek seedlings [15]. Comparative transcriptomic analyses of MeJA-responsive seedlings led to the identification of the key transcription factor *Tf*WRKY40, which positively regulates diosgenin biosynthesis [15,16]. Similarly, in *Dioscorea composita*, *Dc*WRKY11 has been reported to function as a positive regulator of steroidal saponin biosynthesis [17]. In addition, phytosulfokine-α (PSK-α) is a highly conserved sulfated pentapeptide hormone that functions as an important extracellular signaling molecule in plants [18,19,20]. Current studies have primarily focused on its roles in regulating cell proliferation, root development, plant regeneration and responses to environmental stresses [19,21,22,23]. For instance, previous studies have reported that PSK signaling is involved in plant growth regulation, lateral root formation, and developmental processes in legumes [21,24,25,26,27,28]. Although exogenous PSK-α treatment has been reported to affect the accumulation of secondary metabolites such as paclitaxel and hyoscyamine [29,30], the available evidence has largely been derived from cell suspension cultures or in vitro tissue culture systems. To date, little is known about whether PSK-α regulates the biosynthesis of secondary metabolites, including diosgenin, in intact plants, and the underlying molecular mechanisms remain largely unexplored.

Currently, the biosynthesis pathway of diosgenin has been relatively well elucidated. Studies have confirmed that cholesterol is its direct biosynthetic precursor [31]. Cholesterol itself is synthesized from C5 isoprenoid precursors, isopentenyl pyrophosphate (IPP) and dimethylallyl pyrophosphate (DMAPP) [32]. The biosynthesis of diosgenin primarily begins with the cytosolic mevalonate (MVA) pathway, which is derived from acetyl-CoA and catalyzes the formation of IPP and DMAPP [33,34]. 3-Hydroxy-3-methylglutaryl-CoA reductase (HMGR) acts as the rate-limiting enzyme in this process [35]. IPP undergoes a series of enzymatic reactions to form squalene, which is then converted into 2,3-oxidosqualene by squalene epoxidase (SQE) [32,35]. Catalyzed by cycloartenol synthase (CAS), 2,3-oxidosqualene is converted into cycloartenol [32], which is subsequently converted into cholesterol through eight enzymatic steps [36]. Following this, the C22R and C16S positions of cholesterol are sequentially oxidized to generate 16S,22R-dihydroxycholesterol [37]. A subsequent condensation between the hydroxyl groups at C16 and C22 leads to the formation of a furostan-type E-ring intermediate bearing a hydroxyl group at C22 [37,38]. In monocotyledonous *Dioscorea zingiberensis*, this step is catalyzed by two P450 enzymes (*Dz*CYP90B71 and *Dz*CYP90G6), whereas in the dicotyledonous *T. foenum-graecum*, it is catalyzed by a single P450 enzyme (*Tf*CYP90B50) [37]. Finally, the E-ring intermediate is hydroxylated at the C26 position by C26 hydroxylases, such as *Dz*CYP94N8 and *Dz*CYP94D143 from *D. zingiberensis*, and *Tf*CYP72A613 and *Tf*CYP82J17 from *T. foenum-graecum* [37,38]. Subsequently, condensation between the C26 hydroxyl group and the existing C22 hydroxyl group leads to the formation of diosgenin [5,38]. Although the biosynthesis pathway of diosgenin has been largely elucidated, studies on its upstream signaling regulatory mechanisms remain scarce.

PSK-α was first identified in “conditioned medium,” where it was shown to promote the proliferation of low-density cultured cells [22]. Studies in planta have demonstrated that PSK-α signaling primarily enhances root and hypocotyl growth in *Arabidopsis thaliana* by promoting cell elongation [39]. PSK signal transduction is initiated by the specific binding of the ligand to the membrane receptor kinase PSKR, which recruits the co-receptor BAK1/SERK to form an active transmembrane signaling complex [19]. This complex triggers phosphorylation events within the intracellular kinase domains, initiating a signaling cascade that activates second messengers such as cGMP and Ca^2+^, and further transduces the signal to a nuclear transcriptional regulatory network, thereby modulating gene expression in the nucleus [40]. Previous studies have shown that PSK signaling regulates growth and interacts with other hormonal pathways, including brassinosteroid and jasmonate signaling, suggesting that such hormonal crosstalk may contribute to the regulation of secondary metabolism [41]. In tomato, PSK has been shown to significantly promote fruit ripening by inducing phosphorylation of the transcription factor DREB2F [42]. These findings suggest that PSK signaling can regulate downstream transcriptional programs through phosphorylation-dependent mechanisms, raising the possibility that similar signaling modules may also participate in the regulation of specialized metabolism.

This study used *T. foenum-graecum* as the experimental system. Exogenous application of PSK-α, combined with high-performance liquid chromatography (HPLC) analysis, confirmed that PSK-α significantly induces diosgenin accumulation. To uncover the molecular basis of PSK-α-induced diosgenin accumulation, transcriptome sequencing was performed to systematically characterize the key enzyme genes involved in diosgenin biosynthesis, and in PSK signaling under PSK-α treatment. Integrated co-expression analysis identified candidate transcription factors potentially involved in coordinating PSK signaling and diosgenin biosynthesis. This study aims to elucidate the signaling cascade and transcriptional regulatory network underlying PSK-α-mediated regulation of diosgenin biosynthesis. Collectively, this work provides a framework for understanding how peptide hormone signaling coordinates steroidal saponin biosynthesis and provides candidate regulatory components for future metabolic engineering aimed at improving diosgenin biosynthesis.

## 2. Results

### 2.1. PSK-α Promotes Diosgenin Accumulation in Fenugreek

To investigate the effect of PSK-α on diosgenin accumulation in *T. foenum-graecum*, 0.1 μM PSK-α was selected based on its previously reported effectiveness in promoting the accumulation of secondary metabolites such as paclitaxel and hyoscyamine in plant culture systems [29,30]. Diosgenin contents in fenugreek seedlings were subsequently determined at different time points (5, 10, and 15 days) following PSK-α treatment.

**Figure 1:**
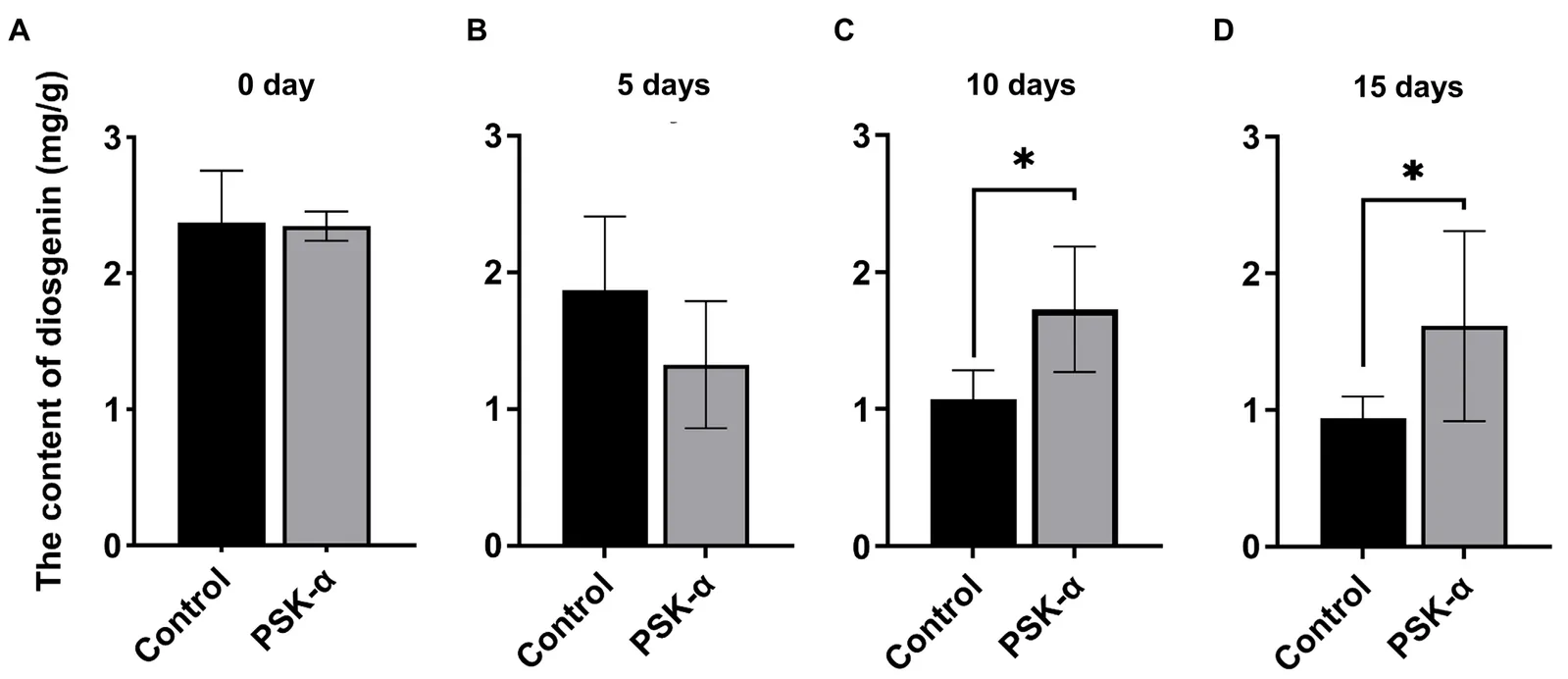
Effects of PSK-α treatment on diosgenin accumulation in fenugreek seedlings. Diosgenin content was determined at 0, 5, 10 and 15 days after treatment with 0.1 μM PSK-α (**A**–**D**). Diosgenin content is expressed as mg/g dry weight. Error bars represent the mean ± standard deviation (SD) of four biological replicates. Statistical significance was determined using Student’s *t*-test. An asterisk (*) indicates a significant difference between the two groups (*p* < 0.05).

Diosgenin contents in all seedling samples were quantified using HPLC, with values ranging from 0.8 to 2.8 mg/g dry weight (DW). At the early stage of treatment (5 days), diosgenin levels in the treated group were slightly lower than those in the control, although the difference was not statistically significant (Figure 1). This mild early decline was only temporary, and metabolite levels displayed distinct dynamic shifts as the treatment duration extended.

Notably, this transient weak reduction at 5 days was followed by a progressive increase in diosgenin accumulation with prolonged PSK-α exposure. After 5, 10, and 15 days of treatment, primary root lengths of the treated seedlings showed an overall increasing trend compared with the control, although the differences were not statistically significant (Figure S1). A clear increase in diosgenin accumulation was observed at the later treatment stages (Figure 1). At 10 days, diosgenin content in the treated group was significantly increased by approximately 61.4% relative to the control (Figure 1). At 15 days, diosgenin content reached 1.6 mg/g DW, representing an approximately 72.1% increase compared with the control (Figure 1).

Overall, these results indicate that exogenous PSK-α promotes diosgenin accumulation in fenugreek seedlings, with the most pronounced effect observed after 15 days of treatment, suggesting PSK-α may be involved in the regulation of diosgenin metabolism.

### 2.2. Transcriptomic Profiling of Fenugreek Seedlings Under PSK-α Treatment

To investigate the temporal transcriptional responses of PSK-α on gene expression involved in diosgenin biosynthesis and PSK signaling pathways, comparative transcriptomic analyses were performed using fenugreek seedlings subjected to two independent PSK-α treatment durations (5 days and 15 days), along with corresponding random pentapeptide-treated control seedlings.

For the 5-day treatment group, the RNA-seq library sequencing was performed, yielding a total of 12.47 Gb of clean data. The clean data volume of each library exceeded 6.03 Gb, and the Q30 ratio of all libraries was greater than 97.97%. In total, 36,502 expressed genes were annotated, consisting of 35,516 annotated known genes and 986 novel genes. A total of 40,876 expressed transcripts were identified, including 35,151 known transcripts and 5725 novel transcripts (Table S1). For the 15-day treatment group, the RNA-seq library sequencing was performed, yielding a total of 38.65 Gb of clean data. The clean data volume of each library exceeded 5.48 Gb, and the Q30 ratio of all libraries was greater than 96.55%. In total, 42,879 expressed genes were annotated, consisting of 41,113 annotated known genes and 1766 novel genes. A total of 49,350 expressed transcripts were identified, including 40,720 known transcripts and 8630 novel transcripts (Table S1).

Quality assessment based on Q20, Q30, GC content and genome mapping efficiency collectively demonstrated that all sequencing datasets from both 5 d and 15 d treatments were of high quality and suitable for subsequent downstream analyses.

**Figure 2:**
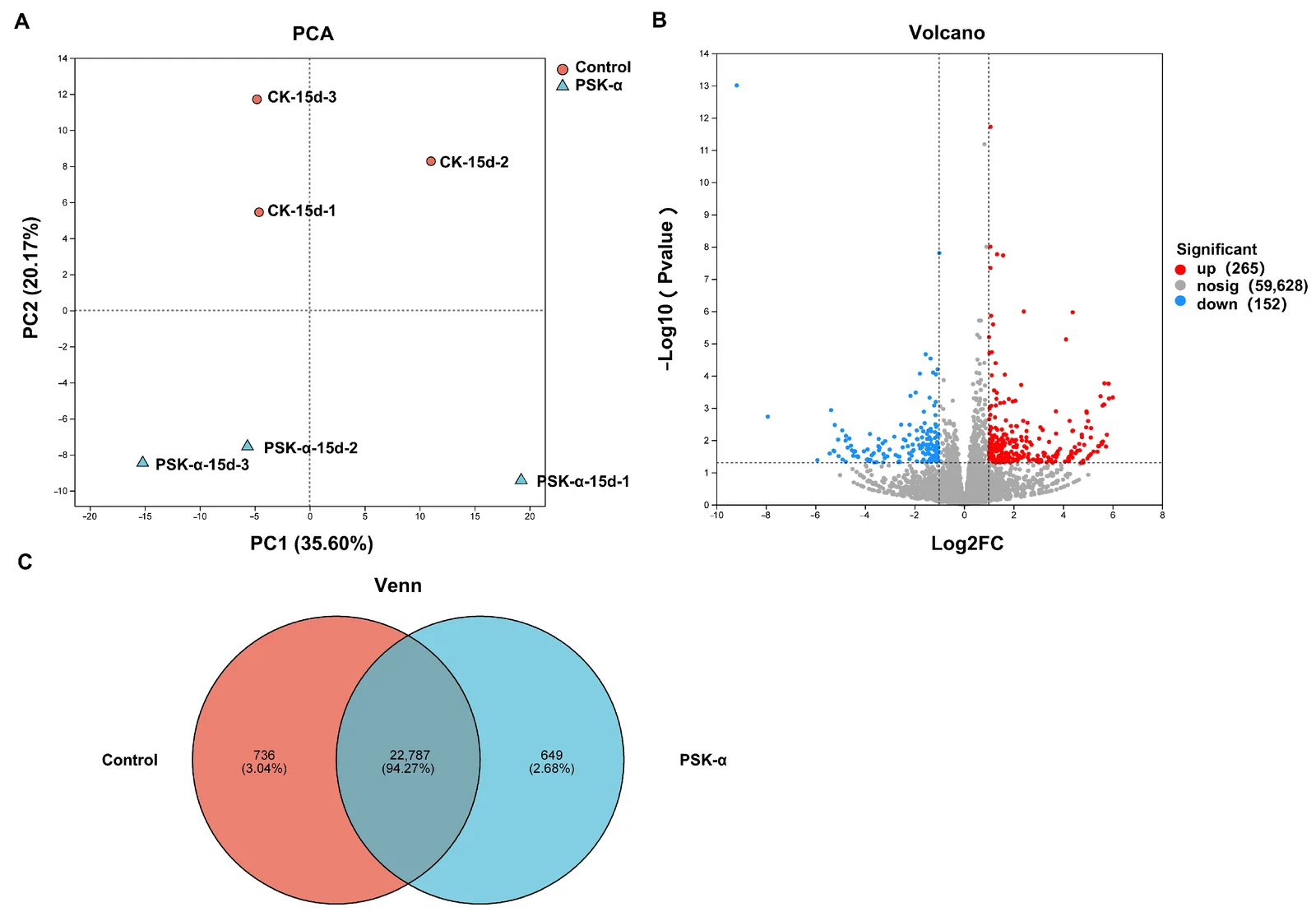
Principal component analysis (PCA) and differential expression analysis of transcriptomic data from seedlings subjected to 15 days of PSK-α treatment. (**A**) PCA plot showing the distribution of samples along the principal component axes (PC1 and PC2). Blue triangles represent the PSK-α-treated group, and red dots represent the control group. (**B**) Volcano plot of differentially expressed genes. (**C**) Venn diagram showing the distribution of expressed genes between the control and PSK-α-treated groups. Vertical dotted lines indicate the log_2_(fold change) cutoff, and the horizontal dotted line indicates the adjusted *P*value cutoff used for identifying differentially expressed genes.

To evaluate the transcriptomic variation among biological replicates after 15 days of PSK-α treatment, principal component analysis (PCA) was performed using the 15-day transcriptomic dataset. The results showed that PC1 and PC2 together accounted for 61.5% of the total variance (Figure 2A), reflecting the major sources of variation among samples. The clear separation between treatment and control groups, together with the high consistency among biological replicates, indicated that the 15-day transcriptomic data were of high quality and suitable for subsequent differential expression analysis.

A total of 60,045 expressed genes were detected in the transcriptome dataset of fenugreek seedlings treated with PSK-α for 15 days, while a total of 35,516 expressed genes were detected in the transcriptome dataset of fenugreek seedlings treated with PSK-α for 5 days. DEGs were identified based on an adjusted *P*adjust < 0.05 and |log_2_FC| ≥ 1, following previously reported criteria [43]. In total, 417 DEGs were identified between the 15-day PSK-α-treated and control groups, of which 265 genes were upregulated and 152 genes were downregulated in response to PSK-α treatment (Figure 2B). In comparison, 815 genes showing differential expression patterns were identified between the 5-day PSK-α-treated and control groups, including 454 upregulated and 361 downregulated genes (Figure S2A). These DEGs were therefore regarded as candidate early PSK-α-responsive genes.

The Venn diagram analysis showed that the majority of genes were commonly expressed between the control and 15-day PSK-α-treated groups, with 22,787 shared genes accounting for 94.27% of the total expressed genes (Figure 2C), and the same trend was observed for the comparison between the control and 5-day PSK-α-treated groups, with 22,086 shared genes accounting for 91.57% of the total expressed genes (Figure S2B). In addition, 736 (3.04%) and 649 (2.68%) genes were specifically expressed in the control and 15-day PSK-α-treated groups, respectively, while 1057 (4.38%) and 977 (4.05%) genes were uniquely detected in the control and 5-day PSK-α-treated groups, respectively. These results indicate that the overall transcriptomic profiles of both comparison groups were highly similar. Genes showing altered expression at 5 days were considered early response candidates because of the pooled RNA-seq design.

Functional annotation of 35,516 unigenes against six public databases (NR, Swiss-Prot, Pfam, eggNOG, GO, and KEGG) resulted in successful annotation of 34,188 unigenes (96.26%) for the 5-day treatment group; meanwhile, that of 41,113 unigenes resulted in successful annotation of 39,568 unigenes (96.24%) for the 15-day treatment group.

**Figure 3:**
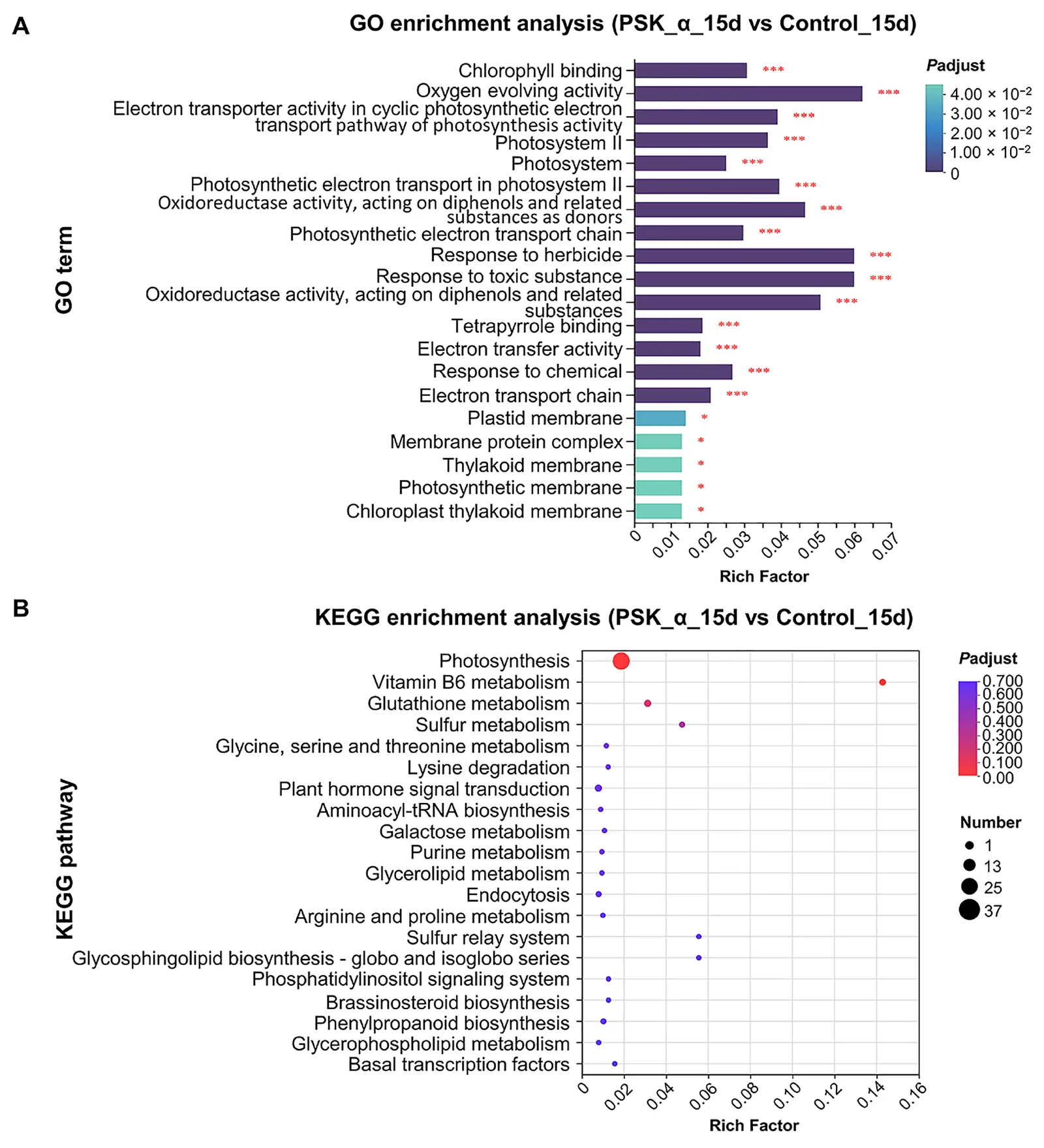
GO and KEGG enrichment analyses of DEGs identified in fenugreek seedlings after 15 days of PSK-α treatment. (**A**) GO enrichment analysis. The y-axis shows GO functional terms, and the x-axis is the Rich Factor, calculated as the ratio of DEGs annotated to a given GO term to the total number of genes annotated to that term. Higher Rich Factor values indicate a greater degree of enrichment. Bar colors represent the adjusted *p* values (*P*adjust), with darker colors indicating greater statistical significance. Red asterisks indicate the significance level of enrichment (* *P*adjust < 0.05 and *** *P*adjust < 0.001). (**B**) KEGG pathway enrichment analysis. The Y-axis shows the enriched KEGG pathways and the X-axis represents the Rich Factor, calculated as the ratio of DEGs annotated to a given pathway to the total number of genes annotated to that pathway. Dot size represents the number of DEGs enriched in each pathway. Dot color represents the adjusted *p* value, with red indicating lower *P*adjust values and greater statistical significance and purple indicating higher *P*adjust values.

GO enrichment analysis of DEGs after PSK-α treatment (Figure 3A and Figure S3A) showed significant enrichment in terms related to “terpene synthase activity,” “sesquiterpenoid biosynthetic process,” and “terpenoid biosynthetic process.” In addition, “oxidation–reduction process” was also significantly enriched, suggesting that a strong secondary metabolic response may have been induced at the cellular level following treatment. Overall, GO analysis indicated that PSK-α treatment mainly affects plant physiological processes by regulating photosynthesis and terpenoid metabolic pathways.

KEGG pathway enrichment analysis further revealed the regulatory effects of PSK-α treatment on the transcriptome of fenugreek seedlings (Figure 3B and Figure S3B). The results showed that the photosynthesis pathway was highly significantly enriched with the highest Rich Factor (adjusted *p* < 0.01), indicating that PSK-α treatment markedly enhanced the expression of photosynthesis-related genes, which may be associated with improved plant growth and energy supply. Pathways related to sesquiterpenoid and triterpenoid biosynthesis, terpenoid backbone biosynthesis, and diterpenoid biosynthesis were also significantly enriched (Figure 3B and Figure S3B), consistent with GO annotation results, suggesting that PSK-α may regulate the terpenoid metabolic network and thereby affect saponin biosynthesis. In addition, significant enrichment of brassinosteroid biosynthesis pathways indicated that PSK-α might modulate the core biosynthesis pathways of steroidal compounds, whose metabolic precursors are shared with diosgenin, thus affecting diosgenin accumulation.

At the level of signal transduction, the plant hormone signal transduction pathway exhibited significant enrichment only in the 5-day treatment group (Figure S3B), whereas no statistically significant enrichment was detected in the 15-day treatment group (Figure 3B). This difference suggested that hormone signaling cascades may exert transient regulatory effects on secondary metabolism at the early stage of PSK-α treatment. Furthermore, stress-responsive pathways including glutathione metabolism and sulfur metabolism were significantly enriched under PSK-α treatment for 5 days (Figure 3B), implying that PSK-α could trigger endogenous plant stress response cascades. Previous studies have shown that environmental stress can induce the accumulation of plant secondary metabolites [44,45]. As a core defensive steroidal metabolite in fenugreek, diosgenin may therefore be positively modulated via the stress signaling cascades activated by PSK-α. These findings provide insights into the potential molecular processes associated with PSK-α-mediated diosgenin accumulation, including stress-responsive signaling pathways.

To further assess the reliability of the transcriptomic data, nine DEGs (*HMGR-3*, *SQE-1*, *CYP90B50*, *PSK3*, *TPST*, *AHA-6*, *bHLH11*, *MYB2*, and *AP2-7)*, representing different expression patterns, were selected for qRT-PCR verification (Figure S4). The qRT-PCR results showed similar expression trends to those obtained from RNA-seq analysis, demonstrating good consistency between the two datasets.

### 2.3. Transcriptional Responses of Diosgenin Biosynthesis-Related Genes to PSK-α Treatment

**Figure 4:**
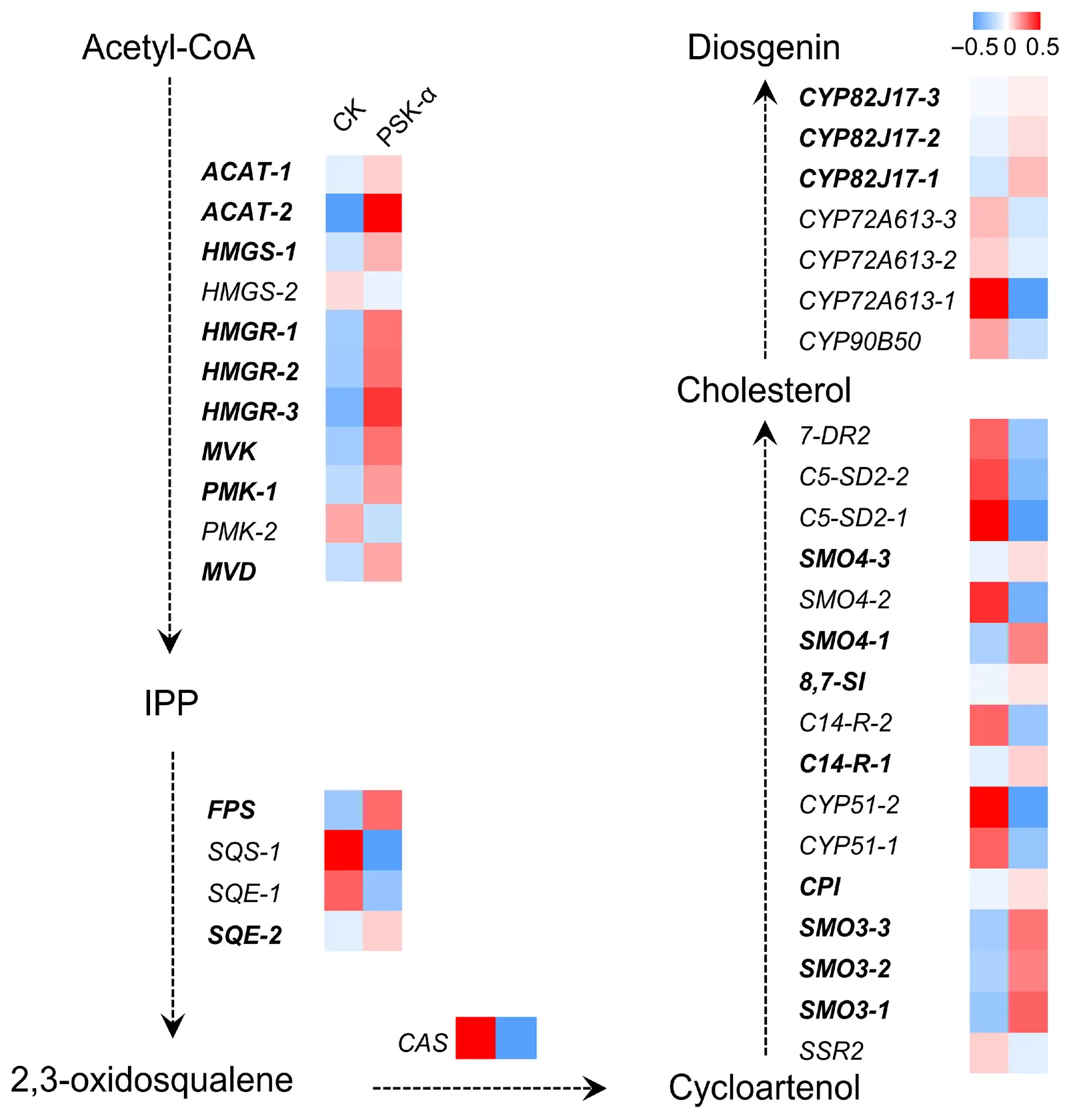
Comparative transcriptomic analysis of key genes or transcript isoforms involved in the diosgenin biosynthesis pathway in fenugreek after 15 days of PSK-α treatment. The heatmap shows the expression patterns of key genes or transcript isoforms associated with the diosgenin biosynthesis pathway. The steroidal saponin biosynthetic route can be broadly divided into three sequential modules: the cytosolic mevalonate (MVA) pathway leading to 2,3-oxidosqualene formation, the cholesterol biosynthesis pathway leading to cholesterol formation from cycloartenol-derived intermediates, and the downstream diosgenin biosynthesis pathway converting cholesterol-related precursors toward diosgenin formation. The dashed arrows indicate multiple consecutive enzymatic reaction steps. In the heatmap, red indicates high gene expression, whereas blue indicates low gene expression. The left side of each heatmap shows the gene or transcript isoform names, and the color scale on the right represents Z-score values. “CK” represents the random pentapeptide-treated group, and “PSK-α” represents the 0.1 μM PSK-α-treated group. Bold font marks transcript isoforms upregulated under PSK-α treatment. The complete transcriptional datasets supporting this analysis are available in Table S2.

The heatmap analysis of the diosgenin biosynthesis pathway visually reflects the regulatory effects of PSK-α treatment on the transcriptional expression of genes involved in diosgenin biosynthesis (Figure 4 and Figure S5). Compared with the control group, 22 transcript isoforms in the biosynthesis pathway were upregulated under PSK-α treatment. In contrast, 15 transcript isoforms were downregulated in the 15-day treatment group (Figure 4). In contrast, 6 transcript isoforms were downregulated and 31 transcript isoforms were upregulated (Figure S5).

Notably, *ACAT-2*, *SQE-2*, *CYP82J17-1* and *CYP82J17-3* exhibited expression trends similar to the changes in diosgenin accumulation after PSK-α treatment of 5 and 15 days (Figure 4 and Figure S5), suggesting that these genes or transcript isoforms may be associated with the transcriptional response accompanying PSK-α-induced diosgenin accumulation.

### 2.4. Analysis of PSK Signaling Pathway Gene Expression in Response to PSK-α Treatment

**Figure 5:**
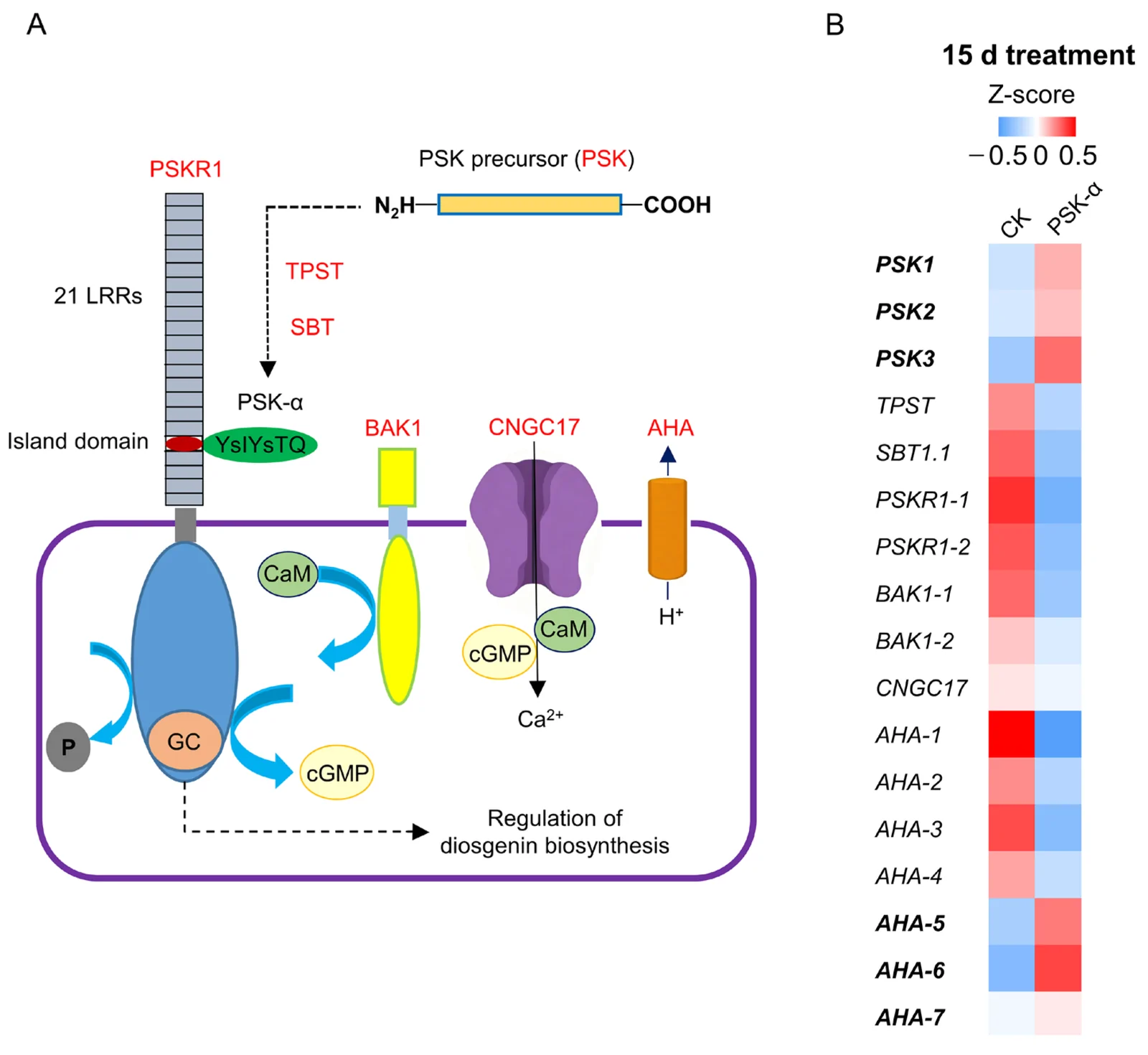
Transcriptional profiling of PSK signaling-associated genes or transcript isoforms under 15-day PSK-α treatment. (**A**) Schematic illustration of the core PSK signaling cascade. The solid curved blue arrows indicate signal transduction and activation of downstream signaling components. Genes marked in red represent pivotal functional components of the PSK signal transduction pathway, including *PSK*, *TPST*, *SBT*, *PSKR1*, *BAK1*, *CNGC17*, and *AHA*, which encode PSK precursor protein, tyrosylprotein sulfotransferase, subtilisin-like serine protease, phytosulfokine receptor 1, BRI1-associated receptor kinase 1, cyclic nucleotide-gated channel 17, and plasma membrane H+-ATPase, respectively. (**B**) Heatmap displaying dynamic transcript profiles in response to 15-day PSK-α treatment, with individual gene expression values scaled by Z-score normalization. Bold font marks transcript isoforms upregulated under PSK-α treatment. The complete transcriptional datasets supporting this analysis are available in Table S3.

To investigate how PSK-α influences endogenous PSK signaling, the expression level of the PSK signaling pathway-related genes was analyzed following PSK-α treatment. Six transcripts showed increased expression trends, whereas 11 transcripts displayed decreased trends under 15-day PSK-α treatment (Figure 5A). By contrast, at the earlier 5-day time point, 4 transcripts showed decreased expression trends, whereas 13 transcripts displayed increased trends under 5-day PSK-α treatment (Figure S6A). AHA family genes are key modulators of extracellular pH homeostasis, which is critical for the proteolytic maturation of precursor PSK peptides and subsequent activation of PSK signal transduction [40,46]. Notably, *AHA-5* was the sole transcript isoform whose expression was consistent with the dynamic accumulation of diosgenin across different treatment time points. Such coordinated changes suggest that PSK maturation and downstream signaling may contribute to the regulation of diosgenin accumulation following PSK-α treatment.

### 2.5. Identification of Key Transcription Factors Regulating Diosgenin Biosynthesis in Response to PSK-α Treatment

**Figure 6:**
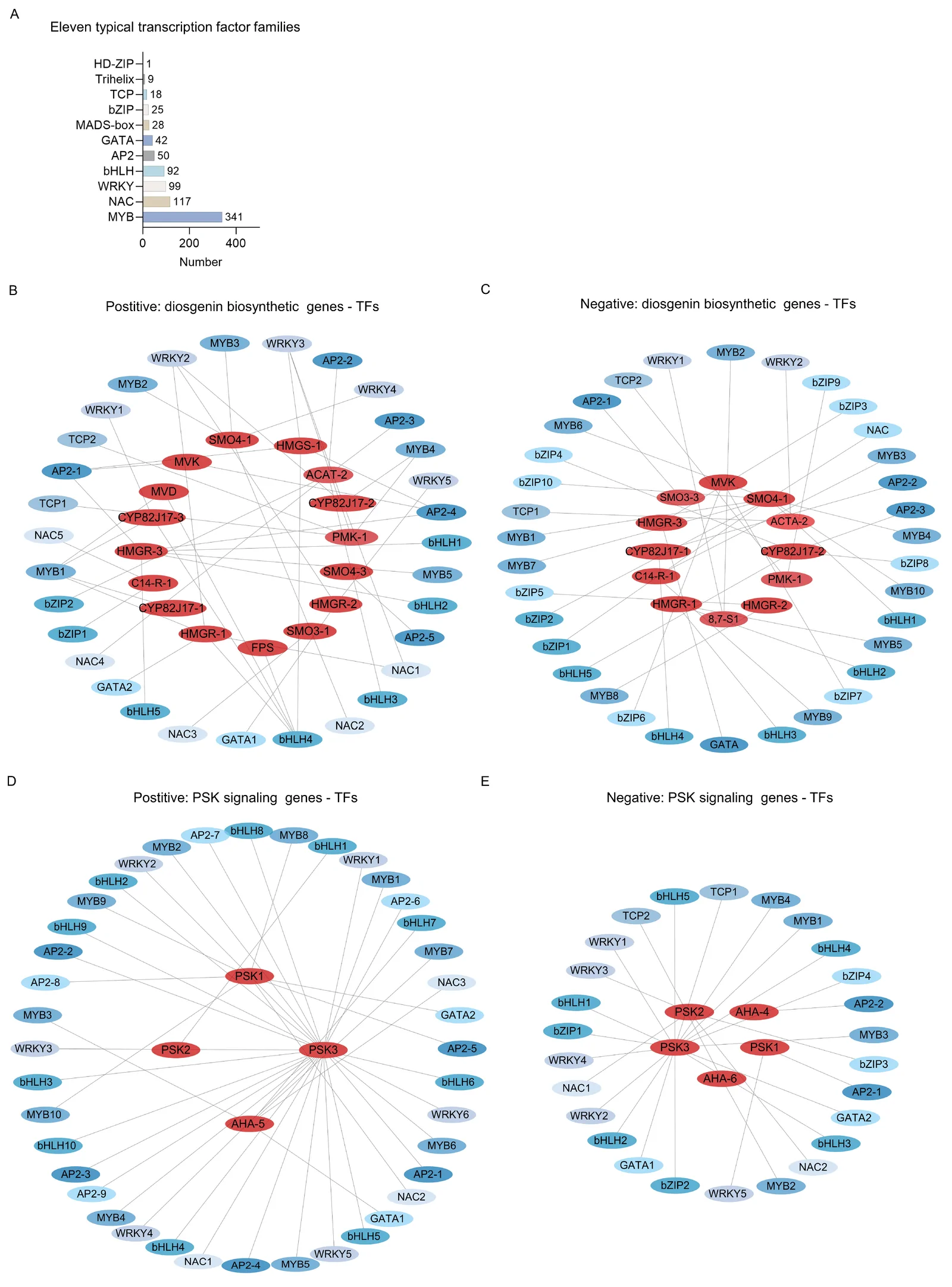
Positive and negative co-expression networks were analyzed for transcription factors (TFs) paired with diosgenin biosynthesis genes and PSK signaling-related genes. (**A**) Overview of the taxonomic distribution of distinct TF families identified in this analysis. (**B**,**C**) Network visualization showing co-expression associations between TF genes and key genes governing diosgenin biosynthesis. (**D**,**E**) Co-expression networks illustrating the regulatory relationships between TFs and PSK signaling pathway genes. Network edges denote statistically validated co-expression interactions (|Pearson *r*| ≥ 0.90 or 0.95, *p* < 0.05), with edge thickness scaled according to Pearson correlation values. Inner nodes denote functional genes belonging to the two target pathways, while outer nodes correspond to various TFs. Node dimensions are proportional to the node connectivity degree within the networks.

Phytosulfokine represents an essential peptide hormone signaling cascade that orchestrates plant growth and specialized metabolism [18,19,20]. Transcription factors are key regulators of plant metabolic processes, particularly in response to changes in plant hormone signaling [47,48]. In this study, a total of 1407 TFs, mainly belonging to 10 typical plant transcription factor families, were identified, including WRKY, MYB, NAC, bHLH, bZIP, MADS-box, HD-ZIP, TCP, GATA, Trihelix (GT factors), and AP2/ERF. Previous studies have shown that members of the WRKY, MYB, and bHLH families are involved in regulating the biosynthesis of terpenoids, alkaloids, and steroidal compounds [16,49,50,51]. Among the total 1407 transcription factors, the MYB family was the most abundant TF family, followed by the NAC, WRKY and bHLH families (Figure 6A).

Given that diosgenin accumulation was markedly enhanced after 15 days of PSK-α treatment, and TF-encoding genes were more comprehensively expressed at this stage, the transcriptomic dataset from 15-day PSK-α-treated fenugreek seedlings was selected for subsequent TF screening. A total of 22 candidate genes related to diosgenin biosynthesis and six core PSK signaling genes were defined as target genes (Table S4). Co-expression analysis was performed between these target genes and 1407 TF-encoding genes. Among the 1407 TF-encoding genes, 32 TFs were positively co-expressed with diosgenin biosynthesis genes (Figure 6B and Table S5), 36 TFs were negatively co-expressed with diosgenin biosynthesis genes (Figure 6C and Table S6), 40 TFs were positively co-expressed with PSK signaling-related genes (Figure 6D and Table S7), and 26 TFs were negatively co-expressed with PSK signaling-related genes (Figure 6E and Table S8). These results suggest that MYB, NAC, WRKY and bHLH TFs may represent candidate regulatory components associated with PSK-α-mediated diosgenin biosynthesis. Other prominent TF families, including AP2/ERF and HD-ZIP, were also highly represented, indicating that they may participate in hormone-mediated signal transduction and the regulation of secondary metabolic pathways under PSK-α treatment.

Considering that PSK signaling is mediated by receptor-like kinases that frequently activate downstream MAPK cascades [52], co-expression analysis was further performed between the total 1407 TF-encoding genes and MAPK signaling pathway genes. First, MAPK pathway genes whose expression patterns were consistent with diosgenin accumulation following 5 and 15 days of PSK-α treatment were used as target genes for the analysis (Table S9). Finally, 16 positively co-expressed transcription factors (Table S10) and 49 negatively co-expressed transcription factors (Table S11) were identified. Among the positively co-expressed transcription factors, only one RGF family member, RGF1, was co-expressed with pathway genes involved in the diosgenin biosynthesis pathway and PSK signaling pathway genes, whose expression was consistent with the dynamic accumulation of diosgenin. Among the negatively co-expressed transcription factors, only one bZIP family member, bZIP29, was co-expressed with pathway genes involved in the diosgenin biosynthesis pathway and PSK signaling pathway genes, whose expression was consistent with the dynamic accumulation of diosgenin. Notably, RGF1 and bZIP29 were the only transcription factors shared among the co-expression networks of diosgenin biosynthesis, PSK signaling and MAPK signaling, suggesting that they may represent candidate regulatory nodes linking PSK signaling with diosgenin biosynthesis.

### 2.6. Prediction of Potentially Regulatory Associations Between Key Transcription Factors and Diosgenin Biosynthesis Genes

To further explore the potential regulatory associations suggested by the co-expression network, candidate TF–target gene pairs were further evaluated based on their predicted interactions with the promoter regions of genes involved in diosgenin biosynthesis. Based on the co-expression relationships, eight transcription factors associated with the key biosynthesis genes *HMGR3* and *SMO4-1* were initially selected as candidate regulators. Among these candidates, those showing the highest correlation coefficients with the corresponding target genes were prioritized for further prediction. Six TF–target gene pairs were ultimately selected, including AP2-1–*HMGR3*, MYB5–*HMGR3*, bHLH1–*HMGR3*, MYB2–*SMO4-1*, MYB3–*SMO4-1*, and WRKY4–*SMO4-1*.

**Figure 7:**
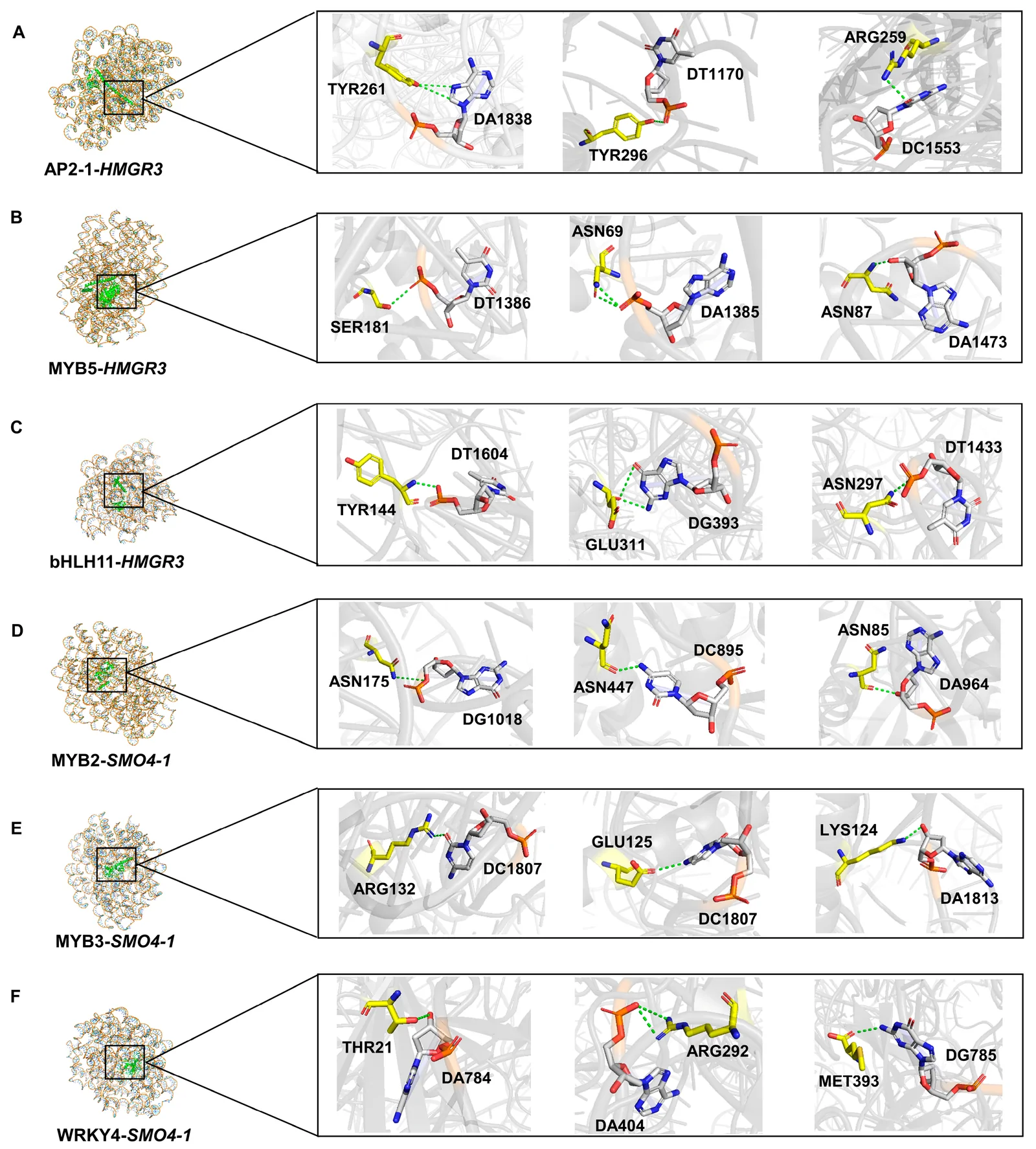
Three-dimensional structures of predicted molecular docking models between key transcription factors and putative target gene promoter regions. The left panels show the overall docking models generated using HDOCK based on the predicted TF structures, whereas the right panels present enlarged views of the predicted interaction interfaces. Green dashed lines indicate predicted hydrogen bonds between interacting residues, and the amino acid residues involved in hydrogen bond formation are labeled. (**A**) AP2-1 docked with the putative *HMGR3* promoter region; (**B**) MYB5 docked with the putative *HMGR3* promoter region; (**C**) bHLH11 docked with the putative *HMGR3* promoter region; (**D**) MYB2 docked with the putative *SMO4-1* promoter region; (**E**) MYB3 docked with the putative *SMO4-1* promoter region; (**F**) WRKY4 docked with the putative *SMO4-1* promoter region.

The predicted docking results showed that all six TFs exhibited potential interaction conformations with the promoter regions of their putative target genes, with hydrogen-bonding interactions contributing to the predicted interface stability (Figure 7). Among the tested TF–target gene pairs, AP2-1–*HMGR3* showed the highest predicted interaction density, with 22 hydrogen bonds identified within the docking interface. These results provide structural insights into the potential associations between candidate transcription factors and diosgenin biosynthesis genes, although further experimental validation is required to confirm these predicted regulatory relationships.

## 3. Discussion

Phytosulfokine-α (PSK-α) is a highly conserved sulfated pentapeptide hormone in plants that has been widely demonstrated to participate in diverse physiological processes, including cell proliferation, organ development, and stress responses [21,46,53,54,55]. Previous studies on PSK-α have primarily focused on its roles in plant growth and development. Only a few reports have suggested that PSK-α can influence the production of secondary metabolites such as paclitaxel and hyoscyamine in plant cell suspension cultures [29,30]. However, its regulatory effects on steroidal secondary metabolites in intact plants and the underlying molecular mechanisms remain largely unexplored. In this study, exogenous application of 0.1 μM PSK-α significantly promoted diosgenin accumulation in fenugreek seedlings after 15 days of treatment. Diosgenin content showed a slight decrease during the early stage of treatment (5 d), followed by a continuous increase during the later stages (10–15 d), ultimately reaching a 72.09% increase compared with the control at 15 d (Figure 1). This biphasic accumulation pattern is distinctly different from the MeJA-mediated diosgenin biosynthetic profile previously observed in fenugreek [15]. Unlike MeJA, which rapidly triggers diosgenin overaccumulation starting at the early treatment phase, PSK-α first induces a transient minor drop in diosgenin before activating robust secondary metabolite synthesis at later time points. This unique temporal response specific to PSK-α may reflect distinct early and late transcriptional responses compared with MeJA. PSK-α may initially trigger transient metabolic adjustments, followed by enhanced activation of diosgenin biosynthetic processes during prolonged treatment.

In untreated control seedlings, the diosgenin content gradually declined over the 15-day culture period (Figure 1). Germinated seedlings exhibited relatively high basal diosgenin levels at 0 days (Figure 1), consistent with the role of steroidal saponins as constitutive defensive metabolites during early seedling establishment [3,56]. In the absence of elicitor treatment such as PSK-α, this intrinsic metabolite pool was progressively utilized to support early vegetative growth, resulting in a continuous decrease in diosgenin levels in control plants. In contrast, PSK-α treatment mitigated this developmental decline in diosgenin accumulation (Figure 1). Although diosgenin levels still decreased at 5 days (Figure 1), likely reflecting the inherent metabolic trajectory of young seedlings, a significantly higher accumulation was observed at days 10 and 15 compared with corresponding controls (Figure 1). These results suggest that PSK-α may contribute to enhanced metabolite accumulation during later developmental stages, potentially through transcriptional changes in diosgenin biosynthesis-related pathways that may partially offset the developmental decline in diosgenin content.

The biosynthesis of diosgenin is initiated from isoprenoid precursors provided by the cytosolic mevalonate (MVA) pathway, followed by construction of the steroidal backbone through the cholesterol biosynthesis pathway, and ultimately formation of the spiroketal structure via multi-site oxidative modifications catalyzed by cytochrome P450 enzymes [32,37,38]. Transcriptomic analysis in this study revealed that, after 15 days of PSK-α treatment, core genes in the MVA pathway and key genes in the cholesterol biosynthesis pathway exhibited a coordinated upregulation pattern, which may contribute to the increased diosgenin accumulation observed at the later stage of treatment (Figure 4). Specifically, within the MVA pathway from acetyl-CoA to 2,3-oxidosqualene, genes or transcript isoforms including *ACAT-1*, *ACAT-2*, *HMGS-1*, *HMGS-2*, *HMGR-1*, *HMGR-2*, *HMGR-3*, *MVK*, *PMK-1*, *PMK-2*, and *MVD* were significantly upregulated (Figure 4). In addition, *FPS* and *SQE-2* were also upregulated. These genes collectively cover the entire MVA pathway from initial substrate activation to the formation of 2,3-oxidosqualene, a common precursor for plant sterols (Figure 4). The availability of 2,3-oxidosqualene is an important factor that may influence the biosynthetic capacity of downstream steroidal metabolites. A previous transcriptomic study has suggested that diosgenin precursors in fenugreek are primarily derived from the MVA pathway, and that MeJA-induced diosgenin accumulation is closely associated with the coordinated upregulation of key MVA pathway genes [15]. Subsequent to the MVA pathway, numerous key enzyme genes or transcript isoforms involved in the synthetic steps from 2,3-oxidosqualene to cholesterol (*SMO3-1*, *SMO3-2*, *SMO3-3*, *CPI*, *C14-R-1*, 8,7-SI, *SMO4-1* and *SMO4-3*), and further from cholesterol to diosgenin (*CYP82J17-1*/*-2*/*-3*), were also significantly upregulated, whose expression trends were consistent with the elevated accumulation of diosgenin (Figure 4). This is highly consistent with the expression patterns observed in the present study under PSK-α treatment, further indicating that PSK-α is associated with coordinated transcriptional responses across multiple steps of the “precursor supply–steroidal backbone formation–terminal modification” pathway.

PSK-α signal transduction is initiated by its binding to the plasma membrane-localized receptor kinase PSKR, which subsequently recruits the co-receptor BAK1/SERK to form an active signaling complex and trigger downstream phosphorylation cascades. [40]. In this study, both *PSKR1*- and *BAK1*-related genes or transcript isoforms (*PSKR1-2*, *BAK1-1* and *BAK1-2*) showed opposite expression trends between the early (5 days) and late (15 days) treatment stages (Figure 5 and Figure S6A). This expression pattern may reflect a transcriptional feedback response associated with prolonged PSK-α treatment. Similar feedback regulation has been widely reported in plant hormone signaling pathways [57,58], where sustained activation of signaling components can lead to the transcriptional repression of receptors or co-receptors to prevent excessive signal amplification and maintain cellular homeostasis. Exogenous PSK-α treatment was associated with altered expression of PSK receptor and co-receptor genes, including *PSKR1-2*, *BAK1-1* and *BAK1-2* (Figure 5A and Figure S6A). The reduced expression of *PSKR1* and *BAK1* genes or transcript isoforms observed after prolonged PSK-α treatment may be consistent with a possible feedback response associated with sustained PSK signaling.

In addition to the PSK signaling pathway itself, the transcriptomic changes observed in the present study suggest potential interactions with other hormone-related regulatory pathways. Previous studies have shown that PSK signaling can interact with brassinosteroid signaling, while PSK has also been reported to act synergistically with MeJA in the regulation of specialized metabolite production [41]. In the present study, the 15-day transcriptomic data revealed significant enrichment of the brassinosteroid biosynthesis pathway following PSK-α treatment (Figure 3B), while plant hormone signal transduction was significantly enriched at the early 5-day treatment stage (Figure S3B). These findings suggest that hormone-related pathways may be involved in the temporal response of fenugreek seedlings to PSK-α treatment. However, the present transcriptomic data do not provide direct evidence that PSK-α interacts with brassinosteroid or jasmonate/MeJA signaling in regulating diosgenin accumulation. Rather, the observed pathway enrichment provides a hypothesis for potential hormonal crosstalk that may contribute to the regulation of steroidal secondary metabolism. Further studies combining hormone measurements, pharmacological treatments, and genetic or molecular validation will be required to determine whether such crosstalk directly contributes to PSK-α-mediated diosgenin accumulation.

Transcription factors are core regulatory components linking upstream hormone signaling with downstream secondary metabolite biosynthesis [48]. In the present study, MYB, NAC, WRKY and bHLH represented the predominant transcription factor families identified in the co-expression networks and exhibited strong associations with both PSK signaling genes and diosgenin biosynthesis genes (Figure 6). These results suggest that members of these TF families may represent candidate components associated with transcriptional responses linking PSK signaling and diosgenin biosynthesis. Both positively and negatively co-expressed transcription factors were identified, indicating that PSK-α-mediated changes in diosgenin metabolism may involve multiple candidate transcriptional regulators rather than a single regulatory pathway.

The molecular docking analysis further evaluated the structural plausibility of the candidate TF–target associations identified through co-expression analysis (Figure 7). The predicted interactions provide structural information that may help prioritize candidate TF–target gene pairs for further investigation. However, molecular docking does not demonstrate that a predicted interaction occurs in vivo, nor does it establish direct DNA binding, transcriptional activation or repression, or a causal effect on diosgenin biosynthesis. Therefore, the docking results should be regarded as supportive and hypothesis-generating evidence rather than definitive evidence of direct transcriptional regulation.

In summary, this study shows that exogenous PSK-α treatment is associated with enhanced diosgenin accumulation in *T. foenum-graecum,* particularly after prolonged treatment. Integrated metabolite profiling and comparative transcriptomic analyses revealed coordinated transcriptional changes involving genes potentially associated with PSK signaling, MAPK signaling, diosgenin biosynthesis, and transcriptional regulation. These findings provide a transcriptomic framework for understanding the molecular processes associated with PSK-α-mediated diosgenin accumulation and expand the potential role of phytosulfokines in plant specialized metabolism. In particular, the coordinated changes observed across diosgenin biosynthesis-related pathways, together with the identification of candidate transcription factors associated with PSK signaling and diosgenin biosynthesis, provide candidate regulatory components for further investigation and may be relevant to future metabolic engineering of diosgenin production.

The findings should nevertheless be interpreted in the context of the evidence provided by the current analyses. Transcriptomic responses and co-expression relationships identify molecular associations but do not by themselves establish direct causal regulation. Accordingly, the candidate transcription factors and their potential regulatory relationships identified in this study represent testable hypotheses that require further experimental validation. Future studies combining promoter-binding assays, genetic manipulation, hormone measurements, and biochemical analyses will be important for determining whether these candidate regulatory components directly contribute to PSK-α-mediated diosgenin accumulation. In addition, analyses of protein abundance and enzyme activities will help determine whether the observed transcriptional changes are accompanied by corresponding changes at the protein and enzymatic levels. A further methodological consideration is that ursolic acid, although suitable as an internal standard under the established HPLC conditions, differs structurally from diosgenin. Potential differences in extraction efficiency and detector response between the two compounds therefore cannot be completely excluded and should be considered when interpreting absolute diosgenin contents. Nevertheless, the same analytical procedure was consistently applied across treatment and control samples, supporting the comparative interpretation of the observed treatment-associated changes in diosgenin accumulation. Further integration of genomic, molecular, and biochemical approaches will help clarify the regulatory mechanisms underlying PSK-α-associated diosgenin accumulation.

## 4. Materials and Methods

### 4.1. Plant Materials, Growth Conditions, and Chemicals

Plant materials used in this study consisted of *T. foenum-graecum* L. seeds originally obtained from a local cultivated population in Anhui Province, China. The seeds were subsequently propagated and maintained in our laboratory for multiple generations, and all experiments were conducted using seeds from the same laboratory-maintained population to ensure consistency of genetic background. Before germination, seeds were stored under identical conditions at 4 °C in the laboratory. Seedlings were cultivated in a controlled-environment chamber under standard growth conditions, including a temperature of 25 ± 2 °C, a 16 h light/8 h dark photoperiod, and 60–70% relative humidity. Diosgenin and ursolic acid, used as the standard chemical, were obtained from YuanYe Bio-Technology Co., Ltd. (Shanghai, China). PSK-α (Tyr(SO_3_H)-Ile-Tyr(SO_3_H)-Thr-Gln) and its scrambled pentapeptide control (Thr-Tyr-Gln-Tyr-Ile) were commercially prepared by Hangzhou DanGang Biotechnology Co., Ltd. (Hangzhou, China). The scrambled pentapeptide, which has the same amino acid composition as PSK-α but a different amino acid sequence, was used as a negative control to account for potential nonspecific effects associated with peptide application.

### 4.2. PSK-α Treatment of Fenugreek Seedlings

Fenugreek seeds of uniform size were selected and surface-sterilized with 75% (*v*/*v*) ethanol for 1 min followed by 5% (*w*/*v*) sodium hypochlorite for 10 min, and then rinsed thoroughly with sterile distilled water. After sterilization, seeds were germinated on 0.65% (*w*/*v*) water agar in darkness at 25 ± 2 °C for 42–44 h, and seedlings with comparable germination status and developmental stage were used for subsequent experiments. PSK-α was dissolved in sterile double-distilled water and added to 1/2-strength Murashige and Skoog (MS) liquid medium to obtain a final concentration of 0.1 μM, while an equimolar scrambled pentapeptide prepared in the same solvent served as the control. Seedlings were then transferred to fresh liquid medium (approximately 30 seedlings per vessel) and supported with sterile glass beads. Each vessel containing approximately 30 seedlings was regarded as one independent biological replicate, and three independent vessels were prepared for each treatment and sampling time point. At 0, 5, 10, and 15 days after treatment, at least 20 seedlings were randomly sampled from each biological replicate for measurement of primary root length and diosgenin determination. Samples collected at 5 and 15 days were additionally used for transcriptomic analysis. Cultures were maintained under a 16 h light/8 h dark photoperiod at 25 ± 2 °C. Samples collected for transcriptomic analysis were immediately flash-frozen in liquid nitrogen and stored at −80 °C, whereas samples used for diosgenin determination were dried at 37 °C to constant weight.

### 4.3. Extraction and HPLC Determination of Diosgenin

The extraction and quantification of diosgenin were performed according to a previously reported method with minor modifications [16,59,60]. Briefly, 20 mg of dried fenugreek seedling powder (constant weight) was accurately weighed, and 6 μL ursolic acid (1 mg/mL) was added as an internal standard. Ursolic acid was selected because it exhibited stable chromatographic behavior, good peak separation, and reliable UV detection at 203 nm under the established HPLC conditions. Moreover, no endogenous ursolic acid peak was detected in fenugreek seedling extracts, indicating that it was suitable for normalization of variations associated with sample preparation and HPLC analysis. Subsequently, 1 mL of methanol was added, and the mixture was subjected to ultrasonic extraction in an ultrasonic bath (250 W, 40 kHz) for 30 min, repeated three times. The combined extracts were evaporated to dryness under a gentle stream of nitrogen.

The residue was reconstituted in 1 mL distilled water, and concentrated sulfuric acid was added to a final concentration of 1.8 M H_2_SO_4_ for acid hydrolysis at 95 °C for 10–12 h. After cooling to room temperature, liquid–liquid extraction was performed twice with n-hexane and once with ethyl acetate to remove non-polar impurities. The organic phases were combined and evaporated to dryness under nitrogen. The residue was redissolved in 100 μL methanol and filtered through a 0.22 μm membrane prior to HPLC analysis.

Diosgenin was separated using a Shimadzu LC-20AT HPLC system equipped with a Shim-pack GIST C18 column (250 × 4.6 mm, 5 μm). Isocratic elution was performed using water (A) and acetonitrile (B) at a ratio of 1:9 (*v*/*v*). The column temperature was maintained at 37 °C, with a flow rate of 0.8 mL/min, an injection volume of 20 μL, and detection wavelength of 203 nm. The total run time was 40 min. Chromatographic data were processed using Shimadzu LabSolutions software (v5.106 SP1). Diosgenin content was quantified using the internal standard method based on calibration curves constructed with authentic standards.

The chromatographic selectivity of the method was evaluated by comparing chromatograms of diosgenin standards, ursolic acid standards, and fenugreek seedling extracts. No interfering peaks were observed at the retention times corresponding to diosgenin and ursolic acid under the established HPLC conditions, indicating satisfactory separation of the target compounds from co-extracted components in the fenugreek matrix.

The analytical performance of the HPLC method was further evaluated through method validation. Calibration curves were established using a series of diosgenin standard solutions (2–20 μg/mL) with ursolic acid (20 μg/mL) as the internal standard (IS). The calibration curve was constructed by plotting the peak area ratio of diosgenin to the internal standard against the corresponding diosgenin concentration (Figure S7). The linear regression equation was Y = 0.02503X + 0.3903, with a correlation coefficient (R^2^) of 0.9968, indicating good linearity within the tested concentration range. The LOD and LOQ were determined based on signal-to-noise ratios of 3 and 10, respectively, and were calculated as 1.57 and 4.77 μg/mL, respectively. The recovery of the internal standard (ursolic acid) during sample preparation was evaluated to assess the consistency of the analytical procedure, with recovery rates ranging from 61.64% to 83.22%. Intra-day and inter-day precision were evaluated using diosgenin standard solutions with ursolic acid as the internal standard and expressed as relative standard deviation (RSD%). The intra-day precision, inter-day precision, and repeatability of the method showed RSD values of 2.38%, 7.26%, and 6.32%, respectively. The identities of diosgenin and ursolic acid peaks were confirmed by comparing their retention times with those of corresponding authentic standards. Representative HPLC chromatograms of diosgenin standards, internal standards, and fenugreek extracts are provided in Figure S8.

### 4.4. Transcriptome Sequencing and Bioinformatic Analysis

Fenugreek seedlings treated with 0.1 μM PSK-α for 5 and 15 days, together with the corresponding controls, were harvested for transcriptome profiling. Three independent biological replicates were used for each treatment, with each replicate consisting of 30 pooled seedlings at 15-day treatment; whereas equal amounts of material from all three biological replicates at 5 d were combined prior to library construction. Total RNA was isolated using the EASYspin Plus Plant RNA Rapid Extraction Kit (Aidlab, Beijing, China), and RNA integrity (RIN ≥ 8.0) was confirmed prior to library preparation. Sequencing libraries were prepared according to standard Illumina protocols and sequenced on the NovaSeq X Plus platform (PE150) by Shanghai Meiji Biomedical Technology Co., Ltd., Shanghai, China.

Raw sequencing reads were filtered by removing adapter sequences, reads containing more than 10% ambiguous bases, and low-quality reads (Q ≤ 20 in more than 50% of bases). Transcript assembly was performed using Trinity [61]. Gene expression levels were quantified using RSEM and expressed as FPKM values. Differential expression analysis was conducted using DESeq2 based on read count data, with thresholds of |log_2_ fold change| ≥ 1 and *P*adj < 0.05. Functional enrichment analyses of DEGs were performed using Gene Ontology (GO) and Kyoto Encyclopedia of Genes and Genomes (KEGG) databases. Volcano plots and heatmaps were generated using GraphPad Prism 9 (Version 9.4.1).

### 4.5. Validation of RNA-Seq Data by Quantitative Real-Time PCR

The selected differentially expressed genes identified from RNA-seq analysis were selected for qRT-PCR validation. RNA samples corresponding to the transcriptome analysis were used for reverse transcription using the TransScript^®^ One-Step gDNA Removal and cDNA Synthesis SuperMix Kit (TransGen Biotech, Beijing, China). All procedures were performed according to the manufacturer’s instructions.

Quantitative real-time PCR was carried out with the Taq Pro Universal SYBR qPCR Master Mix (Vazyme Biotech, Nanjing, China) using a Bio-Rad CFX96^TM^ Real-Time PCR Detection System (Bio-Rad, Hercules, CA, USA). The amplification program consisted of an initial activation at 95 °C for 2 min, followed by 40 cycles of 95 °C for 10 s and 58 °C for 25 s. Fluorescence signals were collected during each amplification cycle. A melting curve analysis was conducted after amplification by gradually increasing the temperature from 65 °C to 95 °C (0.5 °C increments, 5 s per step) to verify the specificity of PCR products. The resulting Cq values were analyzed to determine relative gene expression levels using the 2^−ΔΔCq^ method. Three biological replicates were analyzed, with three technical replicates for each biological sample. The primer sequences used for qRT-PCR analysis are provided in Table S12.

### 4.6. Gene Co-Expression Network Analysis

Pearson correlation coefficients were calculated based on FPKM expression values across all transcriptome samples to identify transcription factors co-expressed with key genes involved in diosgenin biosynthesis, PSK signaling and MAPK signaling pathways. A total of 1407 transcription factor-encoding genes identified from the transcriptome dataset of 15-day PSK-α-treated fenugreek seedlings were included in the analysis. Transcription factors showing strong correlations (|*r*| ≥ 0.90 or 0.95, *p* < 0.01) with the target genes were selected as candidate regulators for further analysis. The co-expression network was visualized using Cytoscape (version 3.10.4).

### 4.7. Molecular Docking Analysis

The three-dimensional structures of selected transcription factors were predicted using AlphaFold 3 through the AlphaFold Server (https://alphafoldserver.com/, accessed on 10 July 2026) [62]. Due to the lack of experimentally characterized promoter sequences in fenugreek, putative promoter regions (~2000 bp upstream of the translation start site) of target genes were predicted based on homologous sequences from *Medicago truncatula*. TF–promoter DNA docking simulations were performed using the HDOCK server (http://hdock.phys.hust.edu.cn/, accessed on 10 July 2026) [63]. The top-ranked docking models were selected for interface analysis using PDBePISA (https://www.ebi.ac.uk/pdbe/pisa/, accessed on 10 July 2026) [64]. The docking conformations and molecular interactions were visualized using PyMOL (Version 3.1) and Discovery Studio Visualizer 2021, respectively.

### 4.8. Statistical Analysis

Data are presented as mean ± standard deviation (SD) from at least three independent biological replicates. Statistical analyses were performed using GraphPad Prism 9 (Version 9.4.1). Differences between two groups were evaluated using Student’s *t*-test. Differences were considered statistically significant at *p* < 0.05 and extremely significant at *p* < 0.01.

## 5. Conclusions

This study provides the first evidence that exogenous PSK-α treatment promotes diosgenin accumulation in *T. foenum-graecum* and is associated with changes in the expression of genes or transcript isoforms involved in diosgenin biosynthesis. Integrated metabolite profiling, comparative transcriptomic analysis, co-expression network analysis, and molecular docking simulations identified candidate genes and transcription factors potentially associated with PSK-α-mediated diosgenin accumulation and further predicted potential TF–target gene interactions. These findings provide a preliminary molecular framework linking peptide hormone signaling with diosgenin accumulation and biosynthesis-related transcriptional responses, and highlight candidate regulatory components for future functional studies and metabolic engineering aimed at improving diosgenin production in fenugreek.

## Data Availability

The transcriptomic data generated and analyzed in this study are available in the NCBI Sequence Read Archive (SRA) repository under BioProject accession number PRJNA1482783.

## Supplementary Materials

The following supporting information can be downloaded at https://www.mdpi.com/article/10.3390/molecules31173046/s1, Figure S1: Effects of 0.1 μM PSK-α treatment on primary root growth of *T. foenum-graecum* (fenugreek) seedlings after 5, 10, and 15 days; Figure S2: Differential expression analysis of transcriptomic data from seedlings subjected to 5 days of PSK-α treatment; Figure S3: GO and KEGG enrichment analysis of differentially expressed genes (DEGs) from fenugreek seedlings with 5 days of PSK-α treatment; Figure S4: Independent qRT-PCR validation of RNA-seq expression patterns of nine representative genes responsive to PSK-α treatment in fenugreek seedlings after 15 days; Figure S5: Comparative transcriptomic analysis of key genes involved in diosgenin biosynthesis pathway in *T. foenum-graecum* after 5 days of PSK-α treatment; Figure S6: Transcriptional profiling of PSK signaling-associated genes under 5-day PSK-α treatment; Figure S7: Calibration curve of diosgenin established by HPLC using ursolic acid as the internal standard (IS); Figure S8: Representative HPLC chromatograms showing the chromatographic separation and selectivity of diosgenin and the internal standard ursolic acid in fenugreek seedling extracts detected at 203 nm; Table S1: Summary statistics of RNA-seq data and de novo transcriptome assembly for *T. foenum-graecum* seedlings treated with PSK-α and control pentapeptide; Table S2: Transcript abundance and functional profiling of diosgenin biosynthesis genes in fenugreek seedlings with PSK-α treatment for 5 and 15 days; Table S3: Transcript abundance and functional profiling of PSK signaling pathway genes in fenugreek seedlings with PSK-α treatment for 5 and 15 days; Table S4: Transcript abundance of target genes selected for co-expression analysis with expression profiles consistent with diosgenin accumulation in *Trigonella foenum-graecum* seedlings under 15-day PSK-α treatment; Table S5: Candidate transcription factors showing positive co-expression with diosgenin biosynthesis genes; Table S6: Candidate transcription factors showing negative co-expression with diosgenin biosynthesis genes; Table S7: Candidate transcription factors showing positive co-expression with genes involved in PSK signaling; Table S8: Candidate transcription factors showing negative co-expression with genes involved in PSK signaling; Table S9: Candidate MAPK cascade genes (marked in red) exhibit transcriptional patterns coordinated with dynamic diosgenin accumulation following 5-day and 15-day PSK-α treatment; Table S10: Candidate transcription factors with positive co-expression correlation to MAPK cascade genes (target genes) in *Trigonella foenum-graecum* seedlings after 15 days of PSK-α treatment; Table S11: Candidate transcription factors with negative co-expression correlation to MAPK cascade genes (target genes) in *Trigonella foenum-graecum* seedlings after 15 days of PSK-α treatment; Table S12: Primer sequences used for qRT-PCR analysis.

## Abbreviations

The following abbreviations are used in this manuscript:

ACAT	Acetyl-CoA acetyltransferase
HMGS	HMG-CoA synthase
HMGR	HMG-CoA reductase
MVK	Mevalonate kinase
PMK	Phosphomevalonate kinase
MVD	Mevalonate diphosphate decarboxylase
FPS	Farnesyl pyrophosphate synthase
SQS	Squalene synthase
SQE	Squalene epoxidase
CAS	Cycloartenol synthase
SSR2	Sterol side-chain reductase 2
CYP51	Sterol C-14 demethylase
C14-R	Sterol C-14 reductase
8,7-SI	Sterol 8,7-isomerase
SMO4	Sterol C4-methyl oxidase 4
C5-SD2	Sterol C-5(6) desaturase 2
7-DR2	7-dehydrocholesterol reductase 2
CYP	Cytochrome P450 monooxygenase
PSKR1	Phytosulfokine receptor 1
TPST	Tyrosylprotein sulfotransferase
SBT	Subtilisin-like serine protease
BAK1	Brassinosteroid insensitive 1 (BRI1)-associated receptor kinase 1
AHA	Plasma membrane H^+^-ATPase
CNGC17	Cyclic nucleotide-gated channel 17
