# Supplementary material for: Comparative Transcriptomic Analysis Provides Insights into PSK-α-Mediated Regulation of Diosgenin Biosynthesis in Trigonella foenum-graecum
Source: Molecules. 2026 Aug 30;31(17):3046. doi: 10.3390/molecules31173046 (PMC13566985; doi:10.3390/molecules31173046)
Supplement: Supplementary file 1 [file molecules-31-03046-s001.zip › molecules-4520117-supplementary.pdf]

Supplementary materials

Comparative transcriptomic analysis provides insights into PSK-α-mediated regulation of diosgenin biosynthesis in *Trigonella foenum-graecum*

Chuanjia Xu [1,2,†], Hao Zhan [1,†], Malakkhanim Mehraliyeva [1], Haoran Liu [1], Mengmeng Zheng [1], Changfu Li [1] and Yansheng Zhang [1,*]

[1] Shanghai Key Laboratory of Bio-Energy Crops, Synthetic Biology Research Center, School of Life Sciences, Shanghai University, Shanghai 200444, China; xuchuanjia@shu.edu.cn (C.X.); 2951167064@shu.edu.cn (H.Z.); malakmehraliyeva@gmail.com (M.M.); liuhaoran001@shu.edu.cn (H.L.); mmz@shu.edu.cn (M.Z.);changfuli@shu.edu.cn (C.L.); zhangys1@shu.edu.cn (Y.Z.)

[2] School of Environmental and Chemical Engineering, Shanghai University, Shanghai 200444, China; xuchuanjia@shu.edu.cn (C.X.)

***Correspondence**: zhangys1@shu.edu.cn (Y.Z.)

†These authors contributed equally to this article.

1. Supplementary Figures

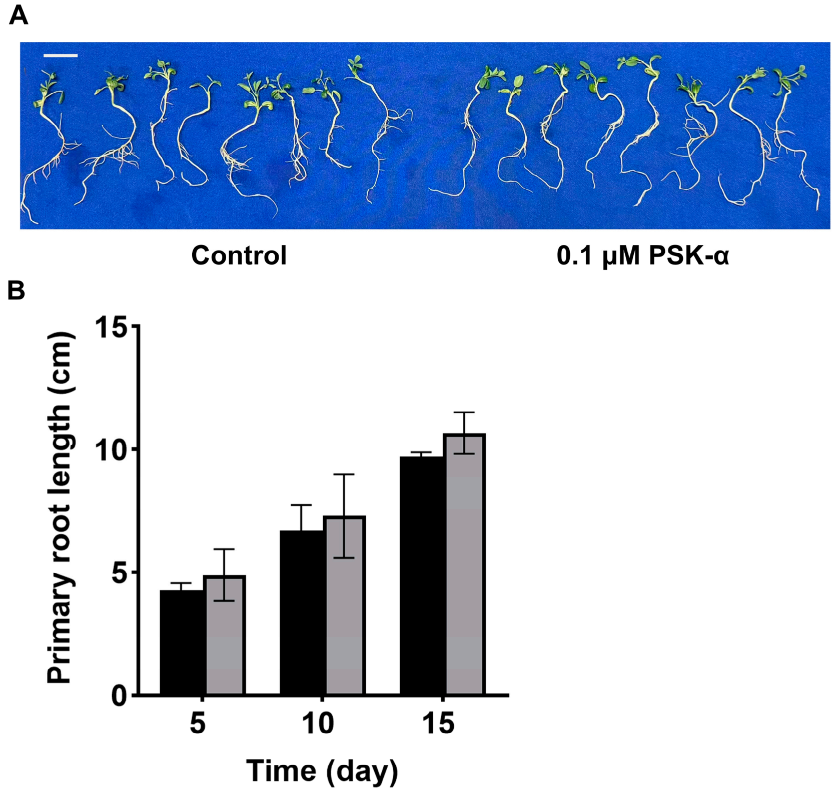

Figure S1. Effects of 0.1 µM PSK-*α* treatment on primary root growth of *T. foenum-graecum* (fenugreek) seedlings after 5, 10, and 15 days. (1) Representative phenotypes of fenugreek seedlings in the control and 0.1 µM PSK-*α* treatment groups after 15 days of treatment. Scale bar = 3 cm. (2) Quantitative analysis of primary root length in control and PSK-*α*-treated seedlings after 5, 10, and 15 days of treatment. Error bars represent the mean ± standard deviation (SD) of measurements from at least 20 independent seedlings. Statistical significance between groups was determined using Student's *t*-test.

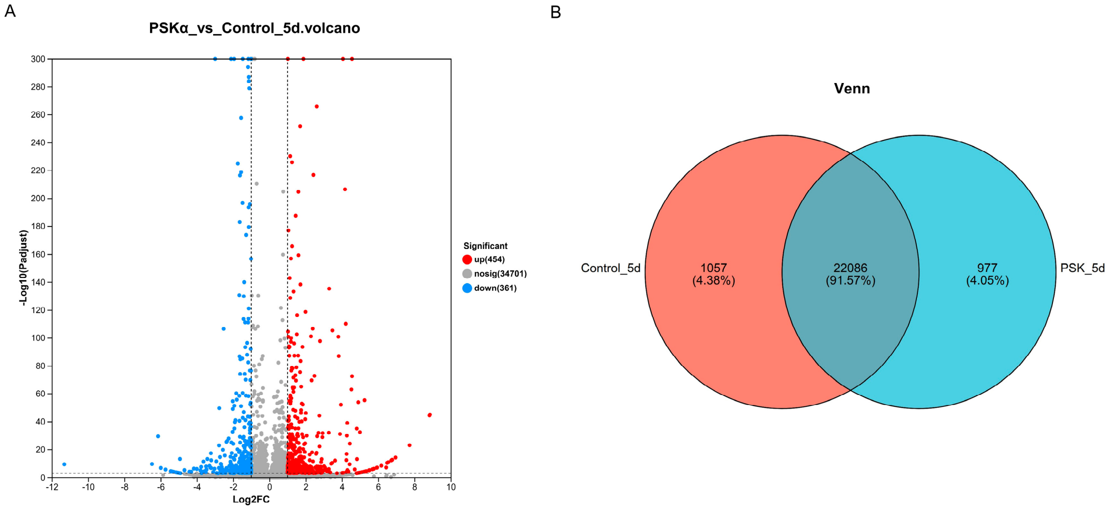

Figure S2. Differential expression analysis of transcriptomic data from seedlings subjected to

5 days of PSK-α treatment. (A) Volcano plot of differentially expressed genes (DEGs). Red dots indicate upregulated genes, blue dots indicate downregulated genes, and gray dots indicate genes that were not significantly differentially expressed. (B) Venn diagram showing the distribution of expressed genes between the control and PSK-α-treated groups. The red region represents genes specifically expressed in the control group, the blue region represents genes specifically expressed in the PSK-α-treated group, and the overlapping region represents genes commonly expressed in both groups.

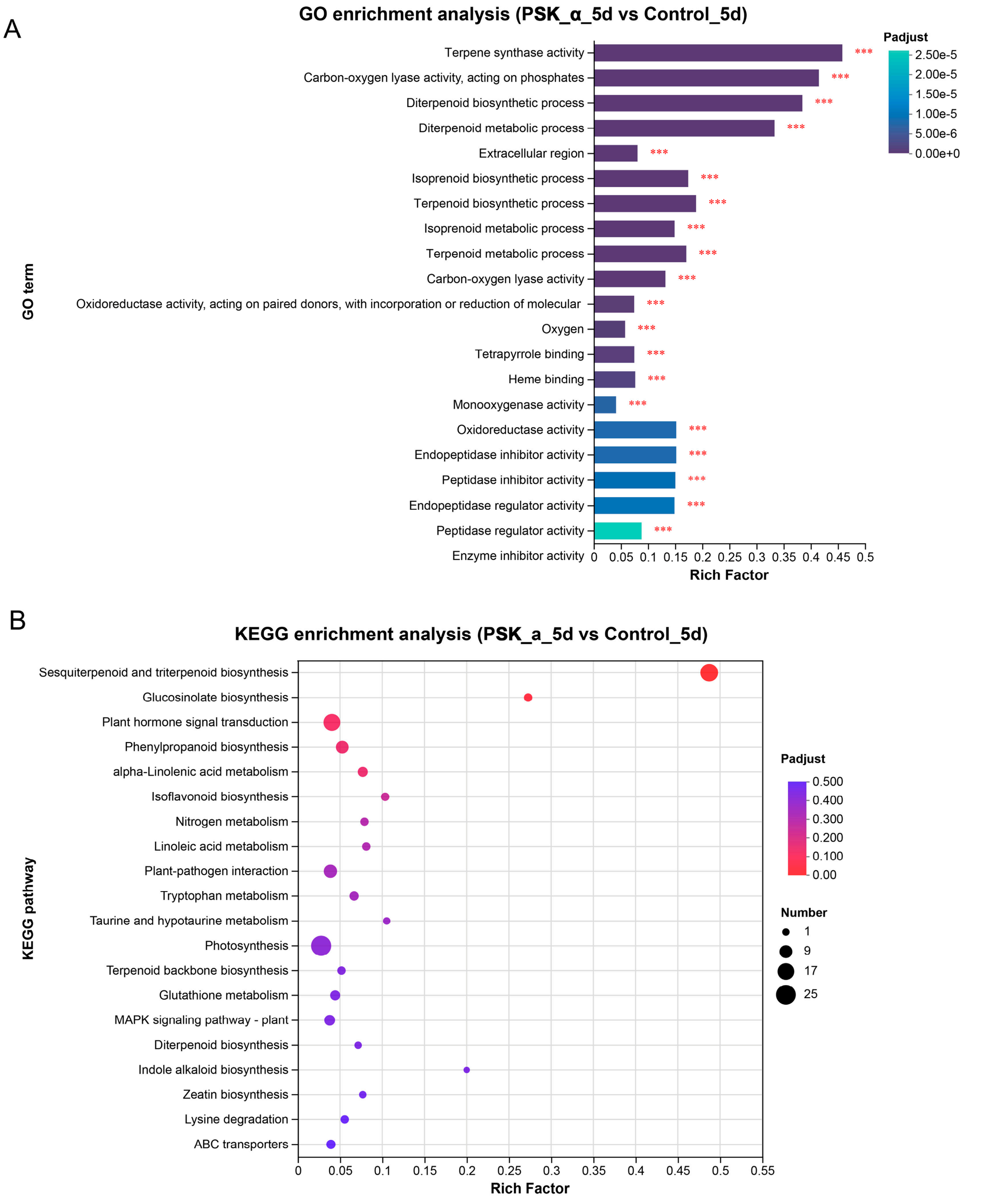

Figure S3. GO and KEGG enrichment analysis of differentially expressed genes (DEGs) from fenugreek seedlings with 5 days of PSK-α treatment. (A) GO enrichment analysis. The vertical axis (GO terms) represents different functional categories. The horizontal axis represents the Rich Factor, where a higher value indicates a greater proportion of DEGs

within the corresponding GO term, indicating a stronger degree of enrichment. Color (adjusted *p*-value, *P*adjust): The adjusted p-value is represented by color intensity. Lighter green indicates a larger *P*adjust value, whereas darker purple indicates a smaller *P*adjust value, corresponding to higher enrichment significance. Significance indicators: *** represents extremely significant enrichment (*P*adjust < 0.001), and ** represents significant enrichment (*P*adjust < 0.01). (B) KEGG pathway enrichment bubble plot. The vertical axis (KEGG pathway) shows the names of significantly enriched pathways of DEGs. The horizontal axis represents the Rich Factor, where higher values indicate a greater proportion of DEGs in the corresponding pathway, reflecting stronger enrichment. Dot color (Pad-just): the adjusted p-value, where redder colors indicate smaller Padjust values and higher enrichment significance, while purpler colors indicate larger Padjust values and lower significance. Dot size (Number): the number of DEGs involved in each pathway; larger dots indicate a higher number of DEGs within the pathway.

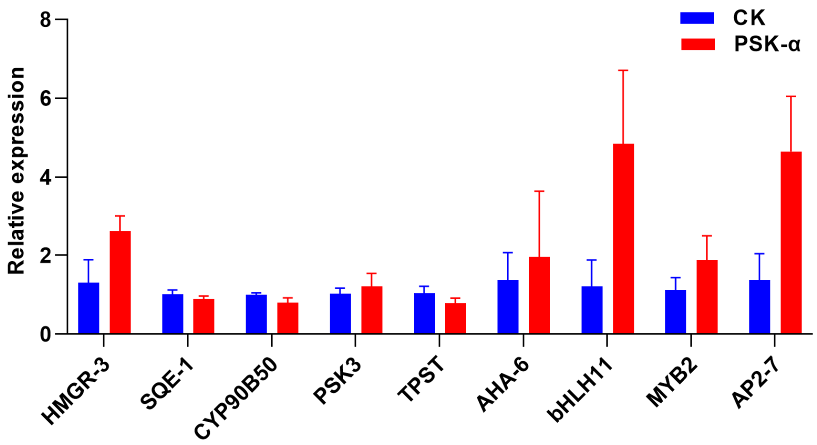

Figure S4. Independent qRT-PCR validation of RNA-seq expression patterns of nine representative genes responsive to PSK-*α* treatment in fenugreek seedlings after 15 days. Relative expression levels were calculated using *TfActin* as the reference gene. Data are shown as means ± SE from three independent biological replicates.

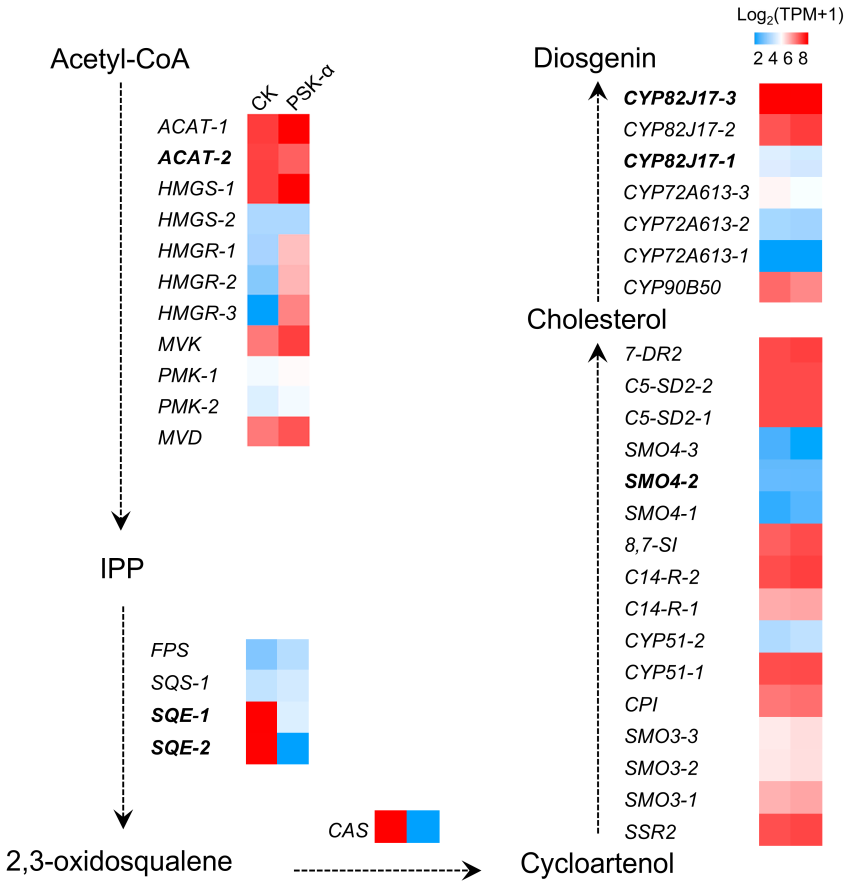

Figure S5. Comparative transcriptomic analysis of key genes involved in diosgenin biosynthesis pathway in *T. foenum-graecum* after 5 days of PSK-*α* treatment. Heatmap of key enzyme gene expression in the diosgenin biosynthetic metabolic pathway following 5 days of PSK-*α* treatment in fenugreek. The pathway includes the mevalonate (MVA) pathway, the cholesterol biosynthesis pathway, and the terminal diosgenin biosynthetic pathway. In the heatmap, red indicates high gene expression, whereas blue indicates low gene expression. The left side of each heatmap shows the gene names, and the color scale on the right represents $\log_2$(TPM+1) values. "CK" represents the random pentapeptide-treated group, and "PSK-*α*" represents the 0.1 μM PSK-*α*-treated group. Bold font marks transcript isoforms downregulated under PSK-*α* treatment.

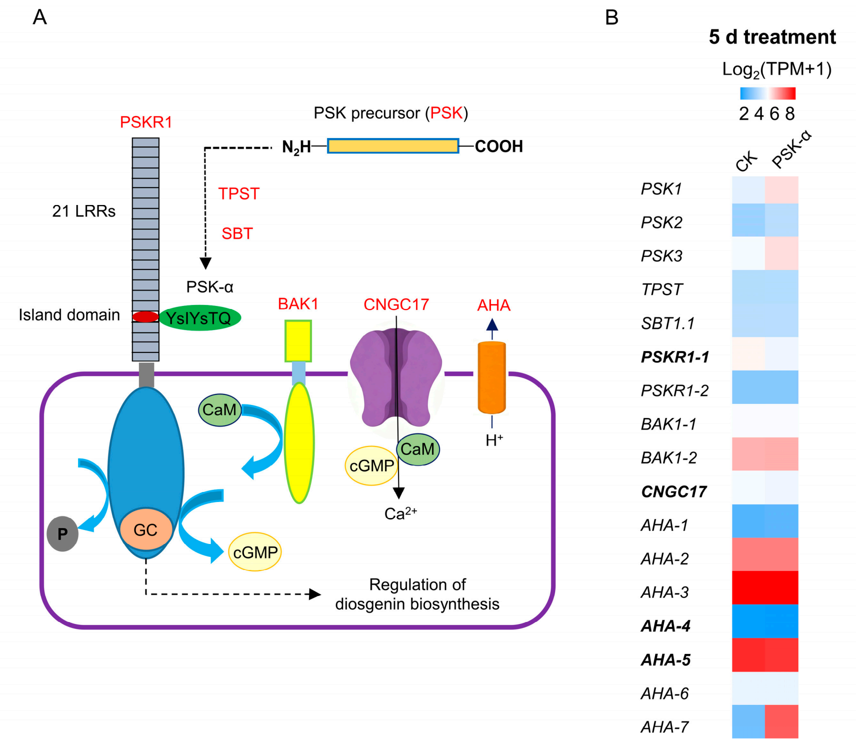

Figure S6. Transcriptional profiling of PSK signaling-associated genes under 5-day PSK-*α* treatment. (A) Schematic illustration of the core PSK signaling cascade. Genes marked in red represent pivotal functional components of the PSK signal transduction pathway, including *PSK*, *TPST*, *SBT*, *PSKR1*, *BAK1*, *CNGC17*, and *AHA*. (B) Heatmap displaying dynamic transcript profiles in response to 5-day PSK-*α* treatment, with individual gene expression values scaled by $\log_2$(TPM+1) normalization. Bold font marks transcript isoforms downregulated under PSK-*α* treatment.

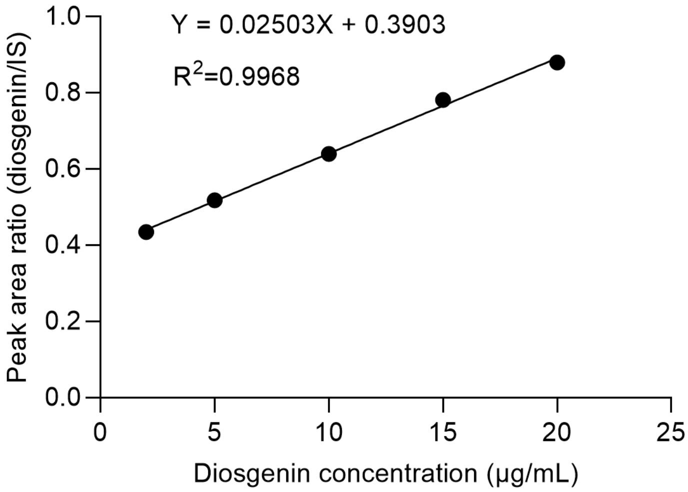

Figure S7. Calibration curve of diosgenin established by HPLC using ursolic acid as the internal standard (IS). The X-axis represents diosgenin concentration (μg/mL), and the Y-axis represents the peak area ratio of diosgenin to the internal standard. The regression equation and correlation coefficient (R^2) are shown in the plot.

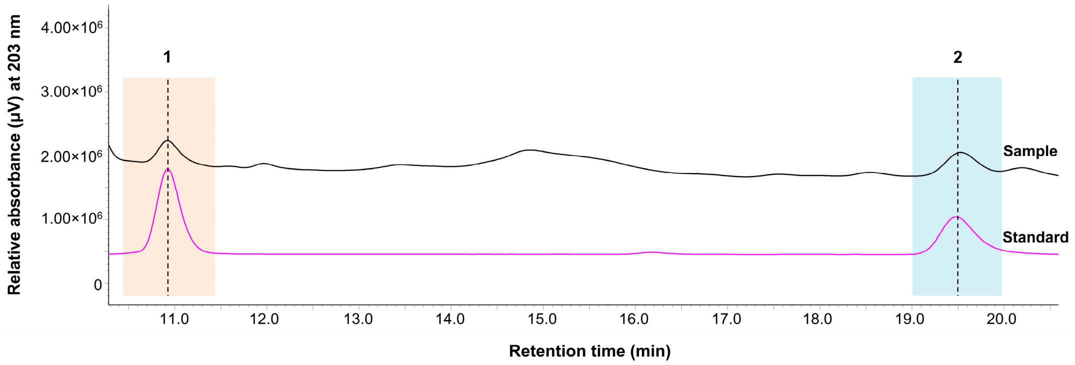

Figure S8. Representative HPLC chromatograms showing the chromatographic separation and selectivity of diosgenin and the internal standard ursolic acid in fenugreek seedling extracts detected at 203 nm. The upper black trace represents the fenugreek sample extract, whereas the lower magenta trace represents the mixed standard solution containing diosgenin and ursolic acid. Peak 1 and Peak 2 indicate ursolic acid and diosgenin, respectively. Shaded areas represent the retention time regions of the corresponding compounds.

2. Supplementary Tables

Table S1. Summary statistics of RNA-seq data and *de novo* transcriptome assembly for *T. foenum-graecum* seedlings treated with PSK-α and control pentapeptide.

Sample name	Raw reads	Raw bases	Clean reads	Clean bases	Q20 (%)	Q30 (%)	GC content (%)
Control_15d_1	44147232	6666232032	43970174	6605118841	99.38	96.66	42.3
Control_15d_2	36649872	5534130672	36491934	5479243614	99.37	96.55	42.11
Control_15d_3	41317900	6239002900	41159198	6185509424	99.39	96.66	42.16
PSK_15d_1	46313676	6993365076	46124008	6929063114	99.38	96.59	41.92
PSK_15d_2	46905604	7082746204	46727338	7012891834	99.39	96.67	42.01
PSK_15d_3	43065428	6502879628	42889096	6434957330	99.37	96.58	42.07
Control_5d	40201220	6070384220	40095120	6034522591	99.63	97.97	41.74
PSK_5d	42843106	6469309006	42727032	6431555008	99.63	98	41.8

Table S2-12 Detailed information is available in the accompanying Excel file.
